# Anillin is required for tumor growth and regulated by miR-15a/miR-16-1 in HBV-related hepatocellular carcinoma

**DOI:** 10.18632/aging.101510

**Published:** 2018-08-09

**Authors:** Yi-Fan Lian, Yan-Lin Huang, Jia-Liang Wang, Mei-Hai Deng, Tian-Liang Xia, Mu-Sheng Zeng, Min-Shan Chen, Hong-Bo Wang, Yue-Hua Huang

**Affiliations:** 1 Guangdong Provincial Key Laboratory of Liver Disease Research, the Third Affiliated Hospital of Sun Yat-sen University, Guangzhou, China; 2 Department of Infectious Diseases, the Third Affiliated Hospital of Sun Yat-sen University, Guangzhou, China; 3 Department of Hepatobiliary Surgery, the Third Affiliated Hospital of Sun Yat-sen University, Guangzhou, China; 4 State Key Laboratory of Southern China, Collaborative Innovation Center for Cancer Medicine, Sun Yat-sen University Cancer Center; Guangzhou, China; 5 Department of Hepatobiliary Surgery, Sun Yat-sen University Cancer Center, Guangzhou, China; * Equal contribution

**Keywords:** ANLN, Hepatocellular carcinoma, Cell growth, Prognostic biomarker, microRNA

## Abstract

Anillin (ANLN) is an actin-binding protein essential for assembly of cleavage furrow during cytokinesis. Although reportedly overexpressed in various human cancers, its role in hepatocellular carcinoma (HCC) is unclear. To address this issue, we confirmed that in 436 liver samples obtained from surgically removed HCC tissues, higher ANLN expression was detected in tumor tissues than in adjacent non-tumor tissues of HCC as measured by immunohistochemistry, quantitative real-time PCR and western blotting. Correlation and Kaplan-Meier analysis revealed that patients with higher ANLN expression were associated with worse clinical outcomes and a shorter survival time, respectively. Moreover, ANLN inhibition resulted in growth restraint, reduced colony formation, and a lower sphere number in suspension culture. Mechanistically, ANLN deficiency induced an increasing number of multinucleated cells along with the activation of apoptosis signaling and DNA damage checkpoints. Furthermore, HBV infection increased ANLN expression by inhibiting the expression of microRNA (miR)-15a and miR-16-1, both of which were identified as ANLN upstream repressors by targeting its 3’ untranslated region. Thus, we conclude that ANLN promotes tumor growth by ways of decreased apoptosis and DNA damage. Expression level of ANLN significantly influences the survival probability of HCC patients and may represent a promising prognostic biomarker.

## Introduction

Hepatocellular carcinoma (HCC) is the most common primary liver malignancy, accounting for approximately 700,000 deaths per year [1]. Current treatment options for HCC are limited and generally ineffective. Curative treatments including liver transplantation and hepatic resection are only eligible for 20% of HCC patients due to late stage diagnosis. Frequent relapse also occurs in HCC patients even after receiving radical surgery. The 5-year overall survival rate of HCC patients, particularly in intermediate stage and advanced stages, remains extremely low [2]. Therefore, better understanding of the molecular mechanism related to liver carcinogenesis and further studies of HCC oncogenes would help to develop new diagnostic and therapeutic strategies.

Anillin (ANLN) is a phylogenetically conserved protein that interacts with cytoskeletal components and their regulators [3]. Generally, ANLN acts as the central organizer at the hub of cytokinetic machinery [4]. During cytokinesis process, ANLN accumulates in the contractile ring and is required for furrow ingression by recruiting several key division-related factors, including F-actin, myosin, septins and CD2AP [5,6]. The lack of ANLN is generally associated with correct assembly of the cleavage furrow but fails to complete cell separation, resulting in altered cell shape with multinucleation that possibly leads to genomic instability [7].

Consistent with its prominent role in cytokinesis, dysregulated ANLN expression has long been observed in the development and progression of various human cancers [8]. A high fraction of nuclear ANLN expression in cancer cells was correlated with a poor prognosis in breast cancer patients. Knockdown of ANLN protein caused an increase in senescent cells with large, poly-nucleated morphology and G2/M phase arrest in breast cancer cells [9,10]. In another study, depletion of ANLN expression in human non-small cell lung cancer cells leads to suppression of cell proliferation and multinucleated cell shape [11]. Interestingly, ANLN downregulation inhibited not only cell proliferation, but also migration and invasion in bladder urothelial carcinoma [12], implying a role of ANLN beyond cell cycle regulation. Furthermore, ANLN was identified as a prognostic biomarker and an increase in ANLN mRNA from normal tissue to malignant disease was observed in breast [9], lung [11], bladder [12,13], gastric [14], colorectal [15], nasopharyngeal [16] and pancreatic cancer [17]. However, the underlying role of ANLN in human HCC has not yet been elucidated.

In contrast to the large number of articles describing ANLN’s downstream effects, limited research has been designed to investigate the mechanism leading to the dysregulation of ANLN in cancer development. Two reports indicated that inhibition of miR-217 or miR-497 expression may be responsible for ANLN overexpression in pancreatic ductal adenocarcinoma and nasopharyngeal carcinoma [16,17], respectively. *In silico* pathway prediction analysis revealed that ANLN could be a Wnt/β-catenin responsive gene in gastric tumor. In breast cancer associated fibroblast, Hippo signaling regulates the expression of several cytoskeletal regulators including ANLN, to help remodel the extracellular matrix [18]. Therefore, considering its great impact on cell cycle regulation and cancer development, it would be necessary to discover the upstream regulator of ANLN expression.

The aim of the present study was to investigate the role of ANLN expression in human HCC. The functional impact and potential prognostic predictive value of ANLN expression in HCC were explored. Moreover, microRNAs (miRs) targeting ANLN mRNA expression were also studied. The present study contributed to an enhanced understanding of ANLN expression for HCC cell growth and provided novel insights into its regulatory mechanism in human cancer.

## RESULTS

### ANLN is highly expressed in hepatocellular carcinoma

**Figure 1:**
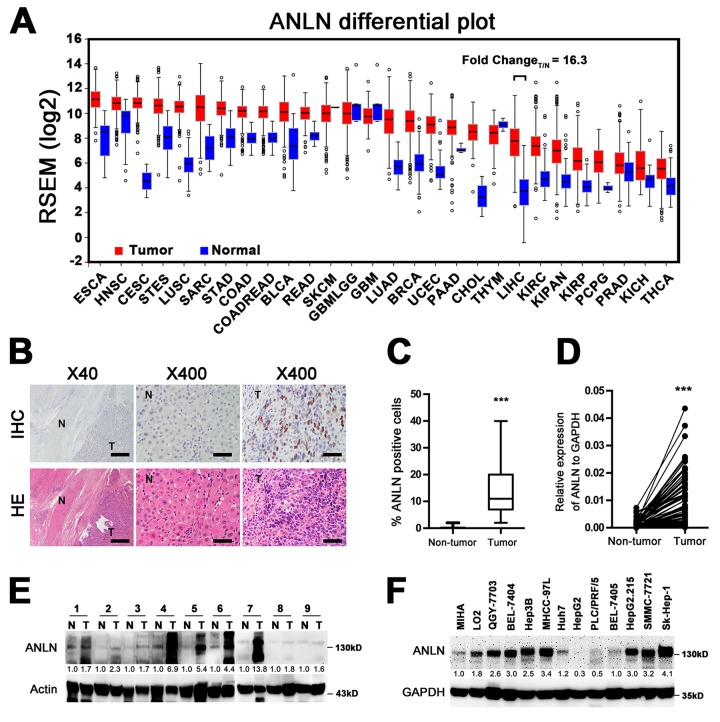
**ANLN is highly expressed in hepatocellular carcinoma.** (**A**) The mRNA levels of ANLN in global human cancer tissues (red) and non-tumor tissues (blue) were analyzed using the TCGA database (http://firebrowse.org/). Noticeably, the fold change of tumor *vs* normal tissue in hepatocellular carcinoma was 16.3. ESCA: Esophageal carcinoma; HNSC: Head and neck squamous cell carcinoma; CESC: Cervical squamous cell carcinoma and endocervical adenocarcinoma; STES: Stomach and esophageal carcinoma; LUSC: Lung squamous cell carcinoma; SARC: Sarcoma; STAD: Stomach adenocarcinoma; COAD: Colon adenocarcinoma; COADREAD: Colon and rectum adenocarcinoma; BLCA: Bladder urothelial carcinoma; READ: Rectum adenocarcinoma; SKCM: Skin Cutaneous melanoma; GBMLGG: Glioblastoma multiforme and brain lower grade glioma (GBM + LGG); GBM: Glioblastoma multiforme; LUAD: Lung adenocarcinoma; BRCA: Breast invasive carcinoma; UCEC: Uterine corpus endometrial carcinoma; PAAD: Pancreatic adenocarcinoma; CHOL: Cholangiocarcinoma; THYM: Tymoma; LIHC: Liver hepatocellular carcinoma; KIRC: Kidney renal clear cell carcinoma; KICH: Kidney chromophobe; KIRP: Kidney renal; KIPAN: Pan-kidney cohort (KICH + KIRC+ KIRP); PCPG: Pheochromocytoma and paraganglioma; PRAD: Prostate adenocarcinoma; THCA: Tyroid carcinoma. (**B**) Representative image of ANLN expression in HCC tissue and matched adjacent tissue by IHC and HE analyses. Scale bar for the left panel: 500 μm; Scale bar for the right panel: 50 μm. T: tumor; N: non-tumor. (**C**) Quantification of the percentage of ANLN-positive cells in tumor tissues and non-tumor tissues from 201 HCC slices. ***P < 0.001. (**D**) The mRNA levels of ANLN from 81 pairs of HCC tissues and matched adjacent non-tumor tissues were tested by quantitative PCR. GAPDH was used as an internal control. ***P < 0.001. (**E**) The protein levels of ANLN in 9 pairs of HCC tissues and matched non-tumor tissues were determined by western blotting assay. The relative fold changes of T compared with N are shown below. T: tumor; N: non-tumor. (**F**) ANLN expression in HCC cell lines. The relative fold changes of all cell lines compared with MIHA are shown below.

ANLN expression was reported to be correlated with cancer development and progression. In order to get a whole image of ANLN expression profile in human cancer, we searched the TCGA database and found that transcription level of ANLN was upregulated in nearly all types of the listed human cancer. Particularly, in hepatocellular carcinoma, the ANLN transcriptional level was at a fold change of 16.3 in tumor *vs* normal tissue (Figure 1A). We then asked if elevated ANLN expression could be seen in our HCC patients. ANLN expression was firstly compared between tumor and adjacent non-tumor tissues in 201 cases of HCC tissue samples. IHC analysis revealed that ANLN showed a nuclear staining pattern. While hardly seen in non-tumor tissue, the ANLN positive cells accounted for 12.5% of total cells in tumor tissue (P < 0.001, Figure 1B and 1C). Additionally, mRNA and protein levels of ANLN were also upregulated in primary cancer tissues compared with those in adjacent non-tumor tissues of 81 and 9 cases of fresh HCC samples, respectively (Figure 1D and 1E). In addition, we analyzed ANLN expression in HCC immortalized hepatic cell lines. The results showed strong expression of ANLN protein in QGY-7703, BEL-7404, Hep3B, MHCC-97L, HepG2.215, SMMC-7721 and Sk-Hep-1 cells (Figure 1F). Taken together, these results indicated that ANLN overexpression is a common feature in human HCC.

### Upregulation of ANLN is associated with a poor prognosis in human HCC

**Figure 2:**
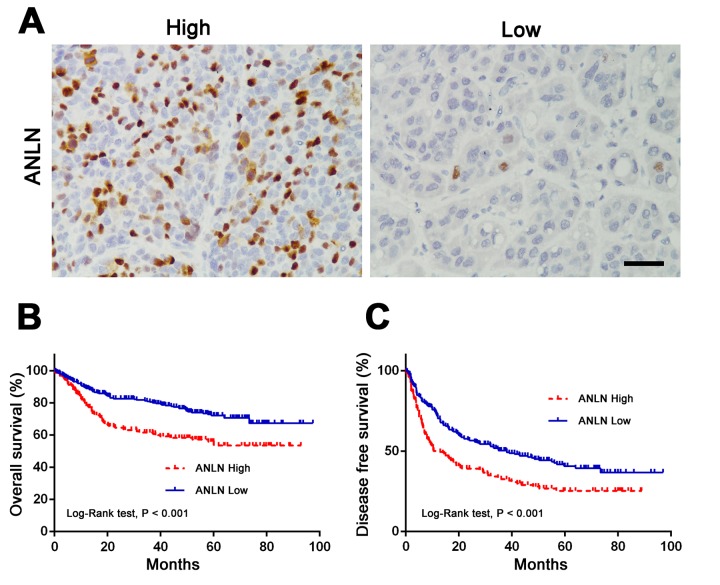
**Upregulation of ANLN is associated with a poor prognosis in human HCC.** (**A**) Representative images of IHC staining of high and low ANLN expression in HCC tissue. Scale bar: 50 μm. (**B**-**C**) ANLN expression was associated with overall survival (**B**) and disease-free survival (**C**) in our HCC cohort according to Kaplan-Meier analysis. ANLN high: 164 samples; ANLN low: 272 samples.

To identify its prognosis predictive value in human HCC, we grouped 436 HCC patients into ANLN expressions of high (n = 164) and low (n = 272) cohorts according to the IHC results (Figure 2A). Correlation analysis was used to examine the association between clinicopathological features and ANLN expression. Results revealed that high ANLN expression had a significant association with a large tumor size (P = 0.008), a high TNM stage (P < 0.001), a high AFP level (P = 0.002), a poor differentiation (P = 0.003) and death events (P = 0.004) (Table 1). Multivariate Cox regression analysis showed that tumor size (HR = 1.104, P < 0.001), tumor thrombus (HR = 2.059, P = 0.019), tumor nodes (HR = 1.821, P = 0.004), ALT level (HR = 1.004, P = 0.006), and ANLN expression (HR = 1.822, P = 0.003) were independently associated with overall survival (Table 2). Moreover, Kaplan-Meier survival analysis indicated that higher ANLN expression was significantly associated with shorter overall survival (P < 0.001, Figure 2B) and disease-free survival (P < 0.001, Figure 2C) in current HCC cohorts. We also acquired survival data from the OncoLnc database [19], which agreed with our results (P < 0.001, Supplementary Figure 1). Taken together, these results suggested that ANLN is an important predictor for prognosis in human HCC.

**Table 1:** Association between ANLN expression and clinicopathological parameters in 436 HCC specimens.

Variable	n	ANLN expression		P
		Low	High	
Age (years)				
≤ 45	164	101	63	0.789
> 45	272	171	101	
Gender				
Male	388	243	145	0.765
Female	48	29	19	
Tumor size (cm)				
≤ 5	170	119	51	0.008*
> 5	262	150	112	
unrecorded	4			
Capsular formation				
Present	86	56	30	0.543
Absent	346	213	133	
unrecorded	4			
Tumor thrombus				
Negative	394	252	142	0.020*
Positive	38	17	21	
unrecorded	4			
Tumor nodes				
single	330	210	120	0.291
multiple	102	59	43	
unrecorded	4			
Ascites				
Negative	405	252	153	0.939
Positive	27	17	10	
unrecorded	4			
Metastasis				
Negative	404	254	150	0.326
Positive	28	15	13	
unrecorded	4			
TMN stage				
I	186	126	60	< 0.001*
II	120	84	36	
III	97	42	55	
IV	26	15	11	
unrecorded	7			
AFP (ng·mL^-1^)				
≤ 400	243	166	77	0.002*
> 400	187	101	86	
unrecorded	6			
HBsAg				
Positive	50	35	15	0.188
Negative	340	205	135	
unrecorded	46			
Cirrhosis				
Negative	126	81	45	0.579
Positive	306	188	118	
unrecorded	4			
ALT (U)				0.461
≤ 40	225	137	88	
> 40	191	123	68	
unrecorded	20			
AST (U)				
≤ 40	213	138	75	0.323
> 40	203	122	81	
unrecorded	20			
ALB (g·L^-1^)				
≤ 35	409	256	153	0.559
> 35	23	13	10	
unrecorded	4			
PT (s)				
≤ 14	373	226	147	0.084
> 14	58	42	16	
unrecorded	5			
Differentiation				
Well	8	7	1	0.003*
Moderate	294	195	99	
Poor	127	64	63	
unrecorded	7			
Prognosis				
Survival	302	202	100	0.004*
Death	134	70	64	

*P < 0.05

Abbreviations: AFP, alpha-fetoprotein; HBsAg, hepatitis B surface antigen; ALT, Alanine aminotransferase; AST, Aspartate transaminase; PT, prothrombin time.

**Table 2:** Univariate and multivariate analysis of overall survival in 436 HCC specimens.

	Univariate analysis				Multivariate analysis		
	Hazard ratio	95% CI	P		Hazard ratio	95% CI	P
Age (years)	0.999	0.985-1.013	0.851				
Gender	1.055	0.625-1.778	0.842				
Tumor size (cm)	1.153	1.106-1.201	< 0.001*		1.104	1.047-1.163	< 0.001*
Capsular formation	0.838	0.54-1.267	0.402				
Tumor thrombus	2.388	1.431-3.985	0.001*		2.059	1.123-3.774	0.019*
Tumor nodes	2.25	1.569-3.227	< 0.001*		1.821	1.205-2.751	0.004*
Ascites	2.013	1.135-3.572	0.017*		1.791	0.974-3.296	0.061
Metastasis	1.937	1.092-3.437	0.024*				
TMN stage	1.487	1.254-1.762	< 0.001*				
AFP (ng·mL^-1^)	1	1.000-1.000	< 0.001*		1	1.000-1.000	0.004*
HBsAg	1.504	0.809-2.795	0.197				
Cirrhosis	1.805	1.174-2.775	0.007*				
ALT (U)	1.002	0.998-1.006	0.322		1.008	1.002-1.014	0.006*
AST (U)	1.004	1.001-1.007	0.018*				
ALB (g·L^-1^)	0.977	0.943-1.011	0.184				
PT (s)	0.991	0.964-1.019	0.513				
Differentiation	0.958	0.673-1.365	0.814				
ANLN expression	1.895	1.347-2.666	< 0.001*		1.822	1.224-2.712	0.003*

*P < 0.05

Abbreviations: AFP, alpha-fetoprotein; HBsAg, hepatitis B surface antigen; ALT, Alanine aminotransferase; AST, Aspartate transaminase; PT, prothrombin time.

### ANLN expression is required for HCC cell growth *in vitro* and *in vivo*

**Figure 3:**
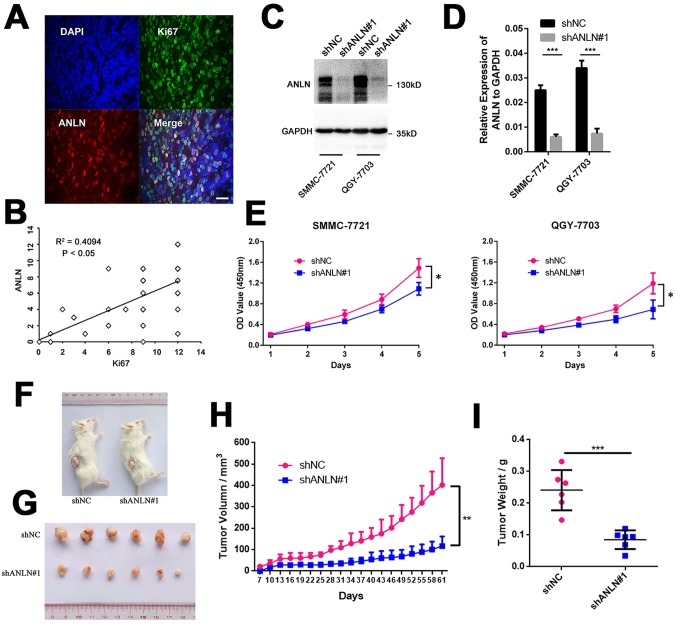
**ANLN expression is associated with HCC cell viability.** (**A**) Representative image of the immunofluorescence analysis of ANLN and Ki67 expressions in HCC tissue. (**B**) Correlation analysis of IHC staining scores for ANLN and Ki67 expressions in 53 HCC slices. (**C**) Western blotting analysis of the knockdown efficacy of ANLN shRNA in SMMC-7721 and QGY-7703 cells. (**D**) Q-PCR analysis of the efficacy of ANLN shRNA in SMMC-7721 and QGY-7703 cells. (**E**) Viability of SMMC-7721 and QGY-7703 cells after ANLN depletion was assessed by MTT assay at indicated times. (**F**) Representative image of NSI mice injected with control and ANLN knockdown SMMC-7721 cells. The red dashed line depicts the border of palpable xenograft tumors. (**G**) All tumors isolated from NSI mice are shown. (**H**-**I**) Growth curves (**H**) and weights (**I**) of xenograft tumors from NSI mice injected with control and ANLN knockdown SMMC-7721 cells. Changes in tumor volumes measured on the indicated days are shown. *P < 0.05; **P < 0.01; ***P < 0.001.

Ki67 is a well-known molecular indicator for cell proliferation. We observed overlapped ANLN expression with Ki67 in HCC tumor tissues by immunofluorescence staining analysis (Figure 3A). The staining scores of ANLN and Ki67 were significantly positively correlated with each other (R^2^ = 0.4094, P < 0.05, Figure 3B). We then constructed ANLN stable knockdown SMMC-7721 and QGY-7703 cells with ANLN-targeting shRNA. ANLN expression was determined by western blotting and qRT-PCR analysis in these two cell lines after lentivirus infection, and the results suggested that ANLN knockdown HCC cell models were successfully established (Figure 3C and 3D). Both SMMC-7721 and QGY-7703 ANLN knockdown cells showed decreased proliferation abilities compared with the control cells *in vitro* as determined by MTT assay (Figure 3E). We also evaluated the effect of ANLN depletion on tumor cell growth *in vivo* by subcutaneously injection of SMMC-7721 control or ANLN knockdown cells into NSI mice. All 6 mice in both groups formed palpable tumors at the injection site (Figure 3F and 3G). In contrast to the control group, mice injected with ANLN-depleted SMMC-7721 cells displayed greatly retarded tumor growth (Figure 3H). Consistently, the average weight of xenograft tumors was also significantly reduced after ANLN depletion (Figure 3I). These results suggested that ANLN downregulation impairs HCC cell growth *in vitro* and *in vivo*.

**Figure 4:**
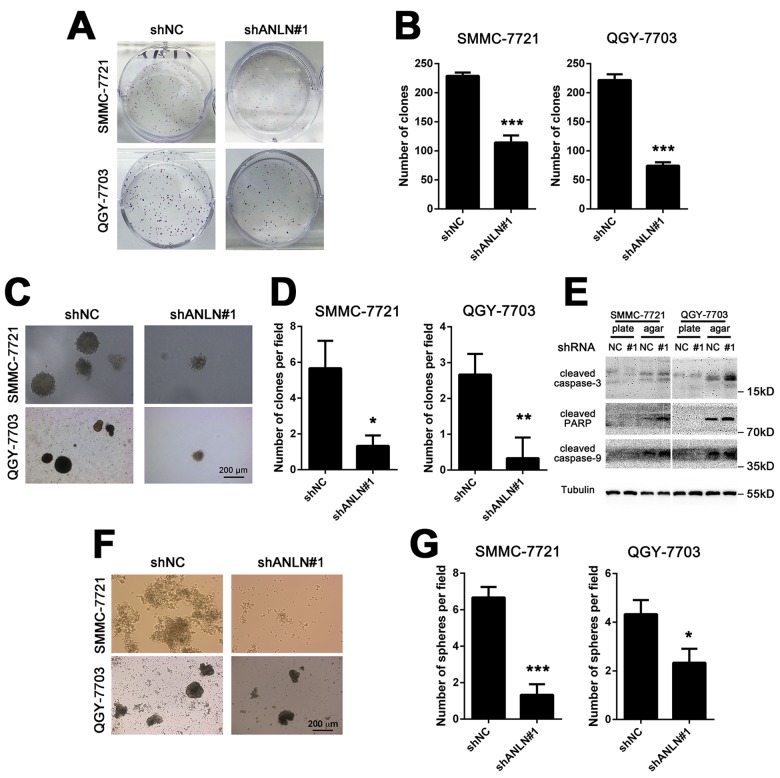
**ANLN expression is required for HCC cell growth.** (**A**) Representative images of colony formation assays of SMMC-7721 and QGY-7703 cells after ANLN depletion. (**B**) Quantification of the colony numbers in (**A**). (**C**) Representative images of soft agar assays of SMMC-7721 and QGY-7703 cells after ANLN depletion. (**D**) Quantification of the colony numbers in (**C**). (**E**) Western blotting analysis of the expression levels of cleaved caspase-3, cleaved PARP and cleaved caspase-9 in ANLN knockdown and control cells derived from plate and soft agar colonies. (**F**) Representative images of the sphere formation assay of SMMC-7721 and QGY-7703 cells after ANLN depletion. (**G**) Quantification of the spherical colony numbers in (f). ***P < 0.001.

Next, we used both cell lines to evaluate how ANLN deficiency affected HCC cell growth. The number of colonies formed in 6-well plates was dramatically decreased after ANLN depletion in both HCC cell lines (Figure 4A and 4B). Data from the soft agar assay showed a 3 to 4-fold decrease in the colony number in ANLN knockdown HCC cells compared with that in the controls (Figure 4C and 4D). We extracted total proteins from cell colonies formed in plates and agar for western blotting. Though hardly detected in the plate, likely due to an inadequate sample amount, ANLN knockdown colonies formed in agar had increasing levels of cleaved caspase-3, cleaved PARP and cleaved caspase-9 compared with the controls (Figure 4E). These results suggested that both anchorage-dependent and -independent growth of HCC cells were heavily inhibited by suppressing ANLN expression. Furthermore, the sphere forming ability was significantly impaired in ANLN-depleted cells compared with that in the controls cells when cultured in suspension, suggesting that ANLN may be involved in the maintenance of stem cell characteristics of HCC cells (Figure 4F and 4G). Therefore, these results clearly indicated that ANLN expression is required for HCC cell growth *in vitro* and *in vivo*.

### ANLN depletion leads to poly-nucleated morphology and DNA damage in HCC cells

**Figure 5:**
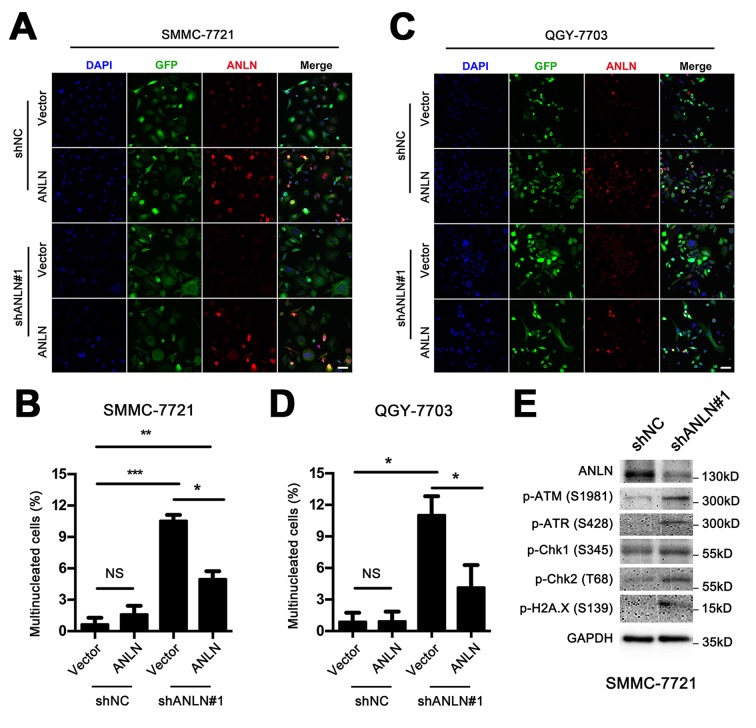
**ANLN depletion leads to poly-nucleated morphology and DNA damage in HCC cells.** (**A**) Representative images of the immunofluorescence analysis of ANLN depletion and reconstitution in SMMC-7721 cells. DAPI stains the nucleus with blue fluorescence and GFP indicates the cell shape with green fluorescence. ANLN is shown in red fluorescence. Scale bar: 50 μm. (**B**) Quantification of the percentages of multinucleated cells in (**A**). (**C**) Representative images of immunofluorescence analysis of ANLN depletion and reconstitution in QGY-7703 cells. (**D**) Quantification of the percentages of multinucleated cells in (**C**). (**E**) Western blotting analysis of the expression levels of phosphorylated ATM (Ser1981), phosphorylated ATR (Ser428), phosphorylated Chk1 (Ser345), phosphorylated Chk2 (Thr68) and phosphorylated Histone H2A.X (Ser139) in ANLN knockdown and control cells.

The lack of ANLN expression is always associated with deficiency during cytokinesis. Previous studies have reported that ANLN depletion leads to an increased number of large, poly-nucleated tumor cells in breast cancer [9,10] and non-small cell lung cancer [11]. Here, we also demonstrated ANLN knockdown induced an increased number of multinucleated cells in HCC cells through immunofluorescence staining. Reconstitution of ANLN expression rescued partly the multinucleated phenotype in both SMMC-7721 (Figure 5A and 5B) and QGY-7703 cells (Figure 5C and 5D). These results indicated that ANLN, at least in part, is required for cytokinesis in HCC cells. Additionally, considering that polyploidy is associated with DNA damage insensitivity and inactivation of pro-apoptotic genes, we tested the expression of several related molecular markers. Noticeably, higher levels of phosphorylated ATM (Ser1981), phosphorylated ATR (Ser428) and their downstream molecules phosphorylated Chk2 (Thr68) and phosphorylated Chk1 (Ser345), respectively, were observed after ANLN depletion. Phosphorylated Histone H2A.X (Ser139) also showed an increasing level in ANLN knockdown cells (Figure 5E). These findings implied that DNA damage could be induced upon ANLN depletion.

### MiR-15a and miR-16-1 mediate the downregulation of ANLN expression in HCC cells

**Figure 6:**
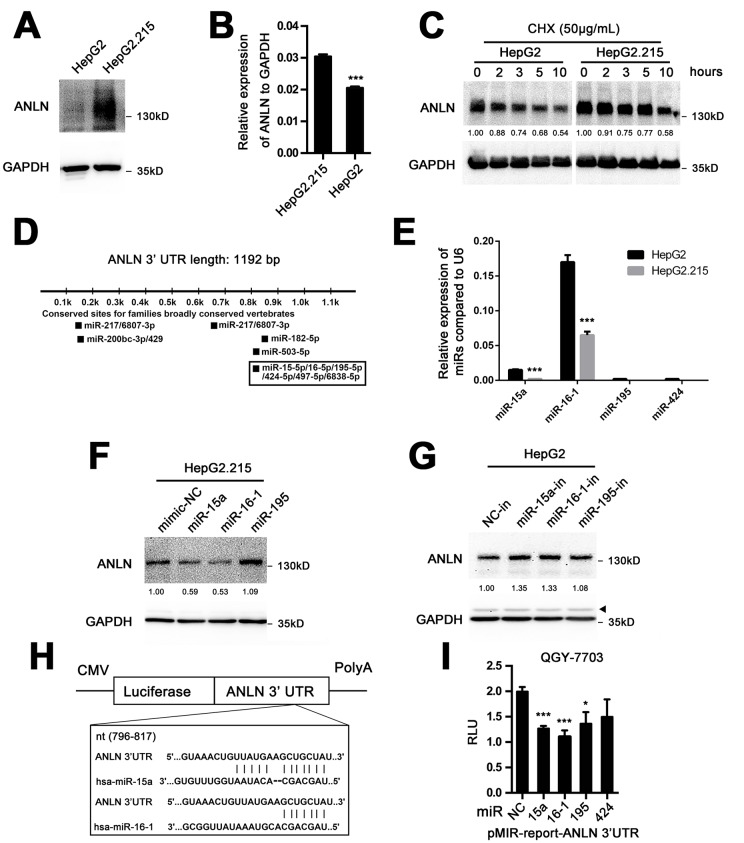
**MiR-15a and miR-16-1 mediate the downregulation of ANLN expression in HCC cells.** (**A**) Western blotting analysis of ANLN protein expression in HepG2 and HepG2.215 cells. (**B**) QPCR analysis of ANLN mRNA expression in HepG2 and HepG2.215 cells. (**C**) Endogenous ANLN protein turnover in HepG2 and HepG2.215 cells over the course of 10 h following the addition of 200 μg·mL^-1^ cycloheximide. GAPDH indicates total protein loading for each sample. The relative fold changes of each sample compared with time 0 are shown below. (**D**) Potential microRNAs targeting full-length ANLN mRNA are shown using the TargetScan tool (www.targetscan.org). (**E**) The endogenous expression levels of miR-15a, miR-16-1, miR-195 and miR-424 in HepG2 and HepG2.215 cells were determined using qPCR. U6 was used as an internal control. (**F**) Western blotting analysis of ANLN expression in HepG2.215 cells treated with miR-15a, miR-16-1, miR-195 and negative control mimics, respectively. The relative fold changes compared with control are shown below. (**G**) Western blotting analysis of ANLN expression in HepG2 cells treated with miR-15a, miR-16-1, miR-195 and negative control microRNA inhibitors, respectively. The asterisk indicates a nonspecific band. The relative fold changes compared with the control are shown below. (H) Schematic diagram of the reporter constructs containing the predicted miR-15a and miR-16-1 binding sites in the 3’ UTR of ANLN. (**I**) Treatment of miR-15a and miR-16-1 mimics significantly attenuated the luciferase activity of the ANLN 3’ UTR compared with the control in QGY-7703 cells. *P < 0.05; ***P < 0.001.

Hepatitis B virus (HBV) infection is the key risk factor for development of HCC in China [20]. The relationship between HBV and ANLN expression in HCC development and tumor progression is unknown. We found that, in HepG2.215 cells, a widely used cell model stably expressing HBV genome, ANLN protein expression was obviously increased compared with that in parental HepG2 cells without HBV genome (Figure 6A). Moreover, ANLN mRNA expression was higher in HepG2.215 cells than in HepG2 cells (Figure 6B). These results provoked our interests to explore whether HBV was involved in ANLN expression and the related underlying mechanism. Firstly, we performed cycloheximide chase analysis of ANLN protein degradation in HepG2.215 and HepG2 cell lines. Data showed that no significant difference was observed in the protein stability observed between these two cells (Figure 6C). We then focused on factors acting at the transcriptional level. Recent reports indicated that HBV RNA could directly inhibit the expression of miR-15a/16-1 in hepatocytes [21,22]. Because microRNAs can target up to 60% of transcribed genes in mammalian cells [23], we performed bioinformatics analysis of microRNAs targeting ANLN transcripts. As expected, both miR-15a and miR-16-1 were possible upstream regulators of ANLN mRNA (Figure 6D). Further analysis confirmed that, in HBV-expressing HepG2.215 cells, both miR-15a and miR-16-1 showed decreased expression levels compared with HBV-negative HepG2 cells (Figure 6E). Addition of either miR-15a or miR-16-1 mimics to the HepG2.215 cells significantly reduced the ANLN mRNA expression (Figure 6F). By contrast, treatment with either the miR-15a or miR-16-1 inhibitor increased ANLN mRNA expression. However, other predicted targeting microRNAs, for example miR-195, had no such effect on HepG2 cells (Figure 6G). Thereafter, the TargetScan algorithm was employed to predict the binding site(s) for miR-15a and miR-16-1 in the 3’ UTR of ANLN mRNA (Figure 6H). To further characterize whether ANLN responds to miR-15a or miR-16-1 through direct 3’ UTR interaction in HCC cells, we cloned a region of the wild-type ANLN 3’ UTR containing the putative miR-15a and miR-16-1 targets into a reporter plasmid downstream of the luciferase gene. The results showed that miR-15a and miR-16-1 mimics led to the attenuation of ﬂuorescence by more than 1.5-fold compared with the negative control (Figure 6I). Altogether, these data indicated that miR-15a and miR-16-1 regulate ANLN expression in HCC cells possibly in an HBV-dependent manner.

## DISCUSSION

In the present study, we demonstrated that ANLN was upregulated in HCC tissues and cell lines. High ANLN level was associated with a poor prognosis in HCC patients. Through shRNA-mediated knockdown cell models, we observed that ANLN depletion resulted in inhibition of cell proliferation and growth, as well as altered cell shape with multinucleation. We also provided evidence that microRNAs including miR-15a and miR-16-1, functioned upstream and regulated ANLN expression in HCC cells. Thus, our study confirmed that ANLN expression contributed to the development and progression of human HCC and may serve as a promising prognostic biomarker.

In fact, elevated ANLN expression was observed in many other human cancers and ANLN expression was proposed to be a prognostic and therapeutic indicator for breast cancer, colorectal cancer, lung cancer and upper urinary tract urothelial carcinoma [8,9,11,12,15]. But little is known regarding ANLN’s role in human HCC. A report using global data mining and multiple reaction monitoring method suggested that ANLN may serve as a potential biomarker when combined with filamin-B (FLNB) to improve the discrimination of HCC patients from the healthy controls [24]. During the submission period of this manuscript, an elegant paper was published by Zhang *et al* concluding that knockdown of ANLN in liver cells inhibits development of liver cancer in MYC-induced, DEN-induced liver tumorigenesis as well as tumor engraftment mouse models [25]. In agreement with the above studies, our results indicated a role of ANLN in the determination of growth rate and progression of human hepatocellular carcinoma. The levels of ANLN were directly correlated with proliferation and transformation of HCC and inversely with survival length. Therefore, current observations suggested that ANLN expression is significantly clinically relevant and might play an essential role in human HCC pathogenesis and development. Additionally, the most unfavorable outcome due to ANLN overexpression supports the hypothesis that direct or indirect destruction of this gene may have a potential therapeutic role in HCC patients.

ANLN protein was first identified as an F-actin binding protein from *Drosophila* embryo extracts [26]. Later, ANLN was found to play a major role in cytokinesis, more precisely in the formation of cleavage furrow at the late stage of mitosis [27,28]. The cell faces a particularly dramatic morphology change during cytokinesis, a process requiring strict regulation of the cytoskeleton [29]. Consistent with the published studies, it was not surprising that an increasing number of large and poly-nucleated cells was observed when ANLN was depleted in our HCC cell models. Although these studies were correlative, they suggested that ANLN depletion led to abnormal cell cycle distribution, with an increase in mitotic cells due to G2/M phase arrest, indicating a prolonged mitotic phase. Failure in cytokinesis may result in genomic instability in two ways: not only by causing genome-destabilizing aneuploid chromosomes but also by promoting the chance of acquiring potentially oncogenic mutations in a prolonged cell cycle [30,31]. Indeed, depletion of ANLN expression in U2OS cells progressively increased the number and intensity of 53BP1 foci in G1 nuclei, a phenomenon representing an increased number of DNA damage events [32]. Therefore, probably by sharing an analogous mechanism, ANLN dysregulation may lead to human cancer from various origins, including HCC.

Importantly, our results indicated for the first time a transcriptional mechanism of ANLN in HBV infected HCC. HBV infection accounts for the most important risk factor of HCC in China, and virus replication is commonly seen in almost all cases of Chinese HCC patients [33]. We observed that ANLN protein was increased in the HBV-positive HepG2.215 cells compared with HBV-negative HepG2 cells, suggesting that HBV may be involved in the regulation of ANLN. Different from the published articles, we identified two novel candidate microRNAs, miR-15a and miR-16-1, which were under the control of HBV replication [21,22] and predicted to targeting the 3’ UTR of ANLN mRNA. Chronic HBV infection has been linked epidemiologically to the development of HCC, although the mechanism is unclear. The virus creates a great impact on the expression of downstream genes that disturb the gene regulatory network in hepatocytes [34]. Our results indicate that HBV might be involved in the regulation of ANLN expression. Nevertheless, whether HBV directly targets ANLN expression or to what extent ANLN expression relies on HBV replication requires further study. Therefore, our investigations reveal several new upstream factors that contribute to ANLN transcriptional regulation in HCC.

The results of the present study were convincing in that upregulation of ANLN expression was observed and predicted a poor prognosis in in human HCC. Although there was a trend showing that ANLN was associated with an aggressive cancer phenotype, an overexpression study is needed to fully demonstrate this point of view because our research was mostly based on the loss-of-function experiments. Additionally, our finding that miR-15a and miR-16-1 could regulate the transcription level of ANLN was preliminary and further analysis is needed.

In conclusion, we identified ANLN as a highly expressed gene in human HCC, and proved that ANLN could serve as a prognostic biomarker for overall survival and disease free survival of HCC patients. We demonstrated that ANLN played an important role in HCC cell growth by regulating proliferation, colony formation and maintenance of proper morphology. In addition, we revealed that microRNAs, including miR-15a and miR-16-1, are as possible upstream regulators for ANLN expression in HCC.

## MATERIALS AND METHODS

### Patients and samples

We obtained a total of 436 paraffin-embedded HCC specimens for prognostic survival analysis from Sun Yat-sen University Cancer Center (Guangzhou, China), among which only 201 cases had both observable tumor tissues and adjacent non-tumor tissues under microscopy. To compare ANLN expression between HCC tissues and adjacent non-tumor tissues and to analyze the relationship between ANLN and Ki67, another 81 fresh HCC specimens were collected from the Third Affiliated Hospital of Sun Yat-sen University (Guangzhou, China). A surgical tumor resection was performed on each patient at department of hepatobiliary surgery. Then tissues were cut into proper size and stored in liquid nitrogen directly for RNA and protein extraction or fixed in 4% paraformaldehyde for immunohistochemistry (IHC). The study was approved by the Institute Research Ethics Committee at the Sun Yat-sen University Cancer Center and the Third Affiliated Hospital of Sun Yat-sen University. Written informed consent was obtained from each patient. Relative experiments with these samples were performed in accordance with the relevant regulations.

### Reagents

Commercially available antibodies were as follows: ANLN (AMAB90662, Sigma), GFP (ab183734, Abcam), α-Tubulin (66031-1-Ig, Proteintech), β-Actin (4967, Cell Signaling Technology), GAPDH (60004-1-Ig, Proteintech), Ki67 (MA5-14520, Invitrogen), cleaved-caspase-3 (9664, Cell Signaling Technology), cleaved-caspase-9 (7237, Cell Signaling Technology), cleaved-poly ADP-ribose polymerase (PARP, 5625, Cell Signaling Technology), phosphorylated ATM (Ser1981) (5883, Cell Signaling Technology), phosphorylated ATR (Ser428) (2853, Cell Signaling Technology), phosphorylated Chk1 (Ser345) (2348, Cell Signaling Technology), phosphorylated Chk2 (Thr68) (2197, Cell Signaling Technology) and phosphorylated Histone H2A.X (Ser139) (9718, Cell Signaling Technology). Noble agar (214220) was purchased from BD Biosciences. All other chemical reagents were obtained from Sigma-Aldrich, unless otherwise indicated.

### Cell culture

293T cells, eleven HCC cell lines (QGY-7703, BEL-7404, Hep3B, MHCC-97L, Huh7, HepG2, PLC/PRC/5, BEL-7405, HepaG2.215, SMMC-7721 and Sk-Hep-1) and two immortalized hepatic cell lines (MIHA and LO2) were employed in this study and were cultured in Dulbecco's Modified Eagle's Medium (DMEM, Invitrogen, Carlsbad, USA) containing 10% fetal bovine serum (FBS, Gibco, Carlsbad, USA) at 37°C and 5% CO_2_. All cell lines were obtained from the College of Life Science, Sun Yat-sen University. Cells were digested and passaged as previously described [35].

### Reverse transcription and quantitative PCR (qPCR)

Total RNA was isolated from tissue specimens and HCC cell lines using Trizol reagent (Invitrogen, Carlsbad, USA) according to the manufacturer’s protocol. Total RNA (1 µg) was reverse transcribed into cDNA by the GoScript™ Reverse Transcription System (Promega, Madison, USA). Quantitative PCR was performed with Platinum SYBR Green qPCR SuperMix-UDG (Invitrogen, Grand Island, USA) with a LightCycler 480 PCR platform (Roche, Indianapolis, USA). Specific primers were as follows: ANLN forward, 5’-GTGATTCTGTTGCTGTCCCG-3’ and ANLN reverse, 5’-GCAGCCTTTTCCTCTGATGG-3’; GAPDH forward, 5’-GGAGCGAGATCCCTCCAAAAT-3’ and GAPDH reverse, 5’-GGCTGTTGTCATACTTCTCATGG-3’. MiR-15a: RT-qPCR stem-loop primer, 5’-GTCGTATCCAGTGCGTGTCGTGGAGTCGGCAATTGCACTGGATACGACCACAAA-3’, qPCR forward primer:5’-GGGTAGCAGCACATAATGG-3’; miR-16-1: RT-qPCR stem-loop primer, 5’-GTCGTATCCAGTGCGTGTCGTGGAGTCGGCAATTGCACTGGATACGACCGCCAA-3’, qPCR forward primer: 5’-GGGTAGCAGCACGTAAATA-3’; miR-195: RT-qPCR stem-loop primer, 5’-GTCGTATCCAGTGCGTGTCGTGGAGTCGGCAATTGCACTGGATACGACGCCAAT-3’, qPCR forward primer: 5’-GGGTAGCAGCACAGAAAT-3’; miR-424: RT-qPCR stem-loop primer, 5’-GTCGTATCCAGTGCGTGTCGTGGAGTCGGCAATTGCACTGGATACGACTTCAAA-3’ qPCR forward primer: 5’-GGGCAGCAGCAATTCATGT-3’; universal reverse primer, 5’-CAGTGCGTGTCGTGGAGT-3’.

### Plasmid construction and transfection

The full-length human ANLN cDNA was amplified from cDNA library of QGY-7703 cells and subcloned into pcDNA3.1(+) (Invitrogen, Carlsbad, USA). Vectors expressing short hairpin RNA (shRNA) against ANLN and scrambled shRNA were kindly gifted from Professor Lunquan Sun [10]. Lentiviruses were produced by co-transfecting constructed plasmids and packaging plasmids psPAX2 and pMD2.G (Addgene, Cambridge, USA) into 293T cells using Lipofectamine 2000 (Invitrogen, Carlsbad, USA) for about 72 h. Culture supernatants were collected, filtered, concentrated and used to infect targeted cells. After 48 h of infection, infected cells were selected by 2 µg·mL^-1^ puromycin (Merck, Darmstadt, Germany) and successful establishment was confirmed by western blotting. The shRNA sequences targeting ANLN mRNA were as follows: shANLN#1, 5’-GCCAATATTCACTACGTATTACTCGAGTAATACGTAGTGAATATTGGCTTTTT-3’. The non-targeting shRNA sequence was served as negative control: shNC, 5’-CTAGCCCGGCCAAGGAAGTGCAATTGCATACTCGAGTATGCAATTGCACTTCCTTGGTTTTTTGTTAAT-3’.

### Cloning of the ANLN 3’ untranslated region (UTR)

To generate the pMIR-Report-ANLN-3’ UTR construct, a region (sense 3599-4720 bp) of ANLN transcript (ENST00000265748.2) containing the predicted targeting microRNAs binding sites was amplified by PCR and cloned into the pMIR-Report luciferase vector (Ambion) between the SpeI and HindIII sites. The PCR primers used were as follows: forward, 5’-GGACTAGTACCGGGAAATTTCCATGCTATCTAG-3’ and reverse, 5’-CCCAAGCTTCCTTTAGACATTTACAGGTATTTATTTGAG-3’. The construct was verified by sequencing.

### Oligonucleotide transfection

The miR-15a, miR-16-1, miR-195, miR-424 mimics and a non-specific miR-control were purchased from Dharmacon. The miR-15a, miR-16-1, miR-195 anti-sense inhibitors and a non-specific miRNA inhibitor control were synthesized by GenePharma. MiRNAs were transfected at a working concentration of 50 nmol·L^-1^ using RNAiMAX reagent (Invitrogen), and oligonucleotide transfection was performed with Lipofectamine 2000 (Invitrogen) according to the manufacturer’s instructions. The transfected cells were incubated at 37°C for 24 h in complete medium and harvested at the indicated time points. TargetScan (http://www.targetscan.org/) was used to predict the ANLN targeting microRNAs.

### Luciferase reporter assay

Approximately 1×10^5^ HCC cells were seeded in 12-well plates and cultured for 24 h. For pMIR-Report related assay, the control vector pRL-TK (Promega) encoding Renilla luciferase, pMIR-Report-ANLN-3’ UTR plasmids and 50 nM each of ANLN targeting microRNA mimics or negative control were cotransfected into cells. Cells were harvested 24 h after transfection. Quantification of firefly and Renilla luciferase activities of at least three independent transfections were measured with the Dual Reporter Assay System (Promega, USA) using FB12 luminometer (Berthold, Germany). The relative luciferase activities (RLU) were calculated by normalizing the activity of the fluorescent luciferase with that of internal standard Renilla luciferase.

### Western blotting

Cells were lysed in NETN buffer (20 mM Tris-HCl at pH 8.0, 100 mM NaCl, 1 mM EDTA, 0.5% Nonidet P-40) containing protease and phosphatase inhibitor cocktail (Thermo Fisher Scientific, Rockford, USA). The lysate protein concentration was measured using the BCA protein assay kit (Pierce, Rockford, USA); after normalization to equal amounts, proteins were separated by 8% or 10% SDS-PAGE, transferred to polyvinylidene fluoride (PVDF) membranes and probed with the indicated primary antibodies. The blots were then incubated with species-specific HRP-conjugated secondary antibodies, and the immunoreactive bands were visualized by enhanced chemiluminescence (ECL, Pierce, Rockford, USA). Quantification of band densitometry was measured with ImageJ software.

### Immunofluorescence analysis

Cells were plated on chamber slides, and fixed with 4% paraformaldehyde at 37°C for 5 min. To keep the stability of microtubule capture at kinetochores, cells were incubated for 5 min on ice before fixation, to destabilize most of the non-kinetochore microtubules. After fixation, cells were permeabilized with 0.1% Triton X-100 for 5 min. Then cells were blocked with 5% BSA for 20 min and incubated with indicated primary antibodies at 4°C overnight. The fluorescence-visualized secondary antibody was added and incubated for 60 min. Nucleus was staining with 50 ng·mL^-1^ DAPI (4',6-diamidino-2-phenylindole, D21490, Invitrogen) for 5 min at room temperature. Fluorescence signal was imaged using a confocal microscope (LSM710, Zeiss). Multinucleated cells were defined as cells with more than one nucleus per cell.

### Proliferation assay

Cell proliferation rate was determined using MTT assay (M6494, Thermo Scientific, Waltham, USA) according to the manufacturer’s protocol. Cells were seeded in 5 replicates in a 96-well plate at a density of 2,000 cells per well and cultured with DMEM containing 10% FBS. Cells were incubated with 20 μL of 5 mg·mL^-1^ MTT for 4h at 37°C. Subsequently, 150 μL of 100% dimethylsulfoxide (DMSO) was added to dissolve the precipitates. Viable cells were counted every day by reading the absorbance at 490 nm with a plate reader (ELx800, BioTek, Winooski, USA).

### *In vivo* animal studies

All the mice were handled according to the Guide for the Care and Use of Laboratory Animals. The procedures were approved by the Institutional Animal Care and Use Committee of Third Affiliated Hospital of Sun Yat-sen University. Female NSI mice [36] (kindly provided by Professor Peng Li from Guangzhou Institutes of Biomedicine and Health, Chinese Academy of Sciences) aged 6 weeks were used for tumor xenografts. All the animals were housed in standard cages (3 animals per cage) under specific pathogen-free conditions. Rodent laboratory chow and tap water were provided ad libitum and maintained under controlled conditions at a temperature of 24 ± 1°C, a humidity of 50 ± 10%, and a 12: 12 h light/dark cycle. Food and water were freely available throughout the experiments. The NSI mice were randomly divided into two groups (4 mice per group), control and ANLN knockdown group, respectively. Cells (5 × 10^6^ in 0.1 mL PBS) were injected subcutaneously into the hind flank of NSI mice. Mice were monitored every 12 h for the first 3 days after inoculation of tumor cells, and then daily thereafter. Tumor sizes were measured every 3 days. Mice were sacrificed 8 weeks post-injection via cervical dislocation, and tumors from two groups were extracted and weighed. The perpendicular diameters of the tumors were measured using a caliper, and the tumor volume was calculated using the following formula: tumor volume (V) = π/6 × large diameter × smaller diameter^2^. The following are general humane endpoints for animals that require euthanasia in this study: 20% decrease in normal body weight; the inability to reach food or water for more than 24 h; a tumor burden greater than 10% body weight or a tumor that exceeds 20 mm in any one dimension. All efforts were made to minimize animal suffering.

### Colony formation assay

Cells were seeded into six-well plates at a density of 2000 per well and incubated at 37°C and at an atmosphere of 5% CO_2_ for 14 days. Additional culture medium was added to the plates at day three. Cells were fixed with methanol, stained with 0.5% crystal violet (C6158, Sigma-Aldrich, St. Louis, USA), and dried. Only clearly visible colonies (more than 50 cells) were counted under a light microscope. The test was repeated three times.

### Soft agar assay

A 2-mL layer of 0.55% agar (wt/vol) in DMEM with 5% FBS was poured into 6-well plates. Cells were re-suspended in 0.33% agar (wt/vol) in DMEM with 10% FBS at a density of 10,000 cells/mL, and 1 mL of the cell suspension was poured on top of the base layer; the suspension was allowed to solidify, followed by incubation at 37°C in 5% CO_2_ for 14 days. The number of colonies was monitored manually under a microscope.

### Sphere formation assay

Sphere formation assays were performed according to previously reported protocols [37]. Briefly, 500 cells were seeded in 6-well plates coated with Ultra-Low Attachment Surface (Corning, New York, USA), followed by culturing for 3 weeks in DMEM/F12 medium (Gibco, Carlsbad, USA) supplemented with B27 (1:50; Gibco, Carlsbad, USA), 20 ng·mL^-1^ EGF (Life Technologies, Carlsbad, USA) and 20 ng·mL^-1^ basic FGF (Life Technologies, Carlsbad, USA). The spheres were observed, imaged and counted under a microscope.

### Immunohistochemistry

IHC was performed as previously described [38]. Briefly, all paraffin-embedded HCC samples were cut into 4-μm sections on a glass slide. Then these slides were dried overnight at 37°C, deparaffinized in xylene twice for 10 min and rehydrated through graded alcohol five times for 5 min, and immersed in 3% hydrogen peroxide for 15 min to block endogenous peroxidase. The sections were boiled in an electric pressure cooker in ethylenediamine tetraacetic acid (EDTA) buffer (pH = 8.0) to retrieve antigen for 3 min. Next, the slides were incubated with 10% normal goat serum at room temperature for 30 min to reduce nonspecific reaction. Sections were then incubated overnight with primary antibody against ANLN at 4°C and anti-rabbit/mouse secondary antibody at room temperature for 1 h. Signals were detected in freshly prepared DAB substrate solution at room temperature for 5 min. Finally, the sections were counterstained with Mayer’s hematoxylin, dehydrated, and mounted. Each section was evaluated by two independent pathologists blinded to the clinical status of patients and graded as described, according to the positive staining intensity (no staining, 0; weak staining, 1; moderate staining, 2; strong staining, 3 scores) and the expression extent scores (< 25%, 1; 25-50%, 2; 50-75%, 3; > 75%, 4 scores). A final immunoreactivity score (IRS) was defined as the intensity score multiplied by the extent score. All scores were subdivided into two categories according to a cutoff value in the study cohort: ANLN high expression (> 7.5) and low expression (< 7.5).

### Statistical analysis

The SPSS software version 20.0 and GraphPad Prism 5 software were used to perform statistical analyses. Correlation of the ANLN staining intensity with clinicopathological characteristics was measured using Pearson’s Chi-Square or Fisher’s exact test. The Cox proportional hazards model and Kaplan-Meier analysis were employed for survival analysis. Spearman’s correlation coefficient was employed for ANLN and Ki67 correlation analysis. The significance of variances between groups was determined by t-test. Each experiment was performed three times in triplicate. Unless otherwise indicated, all error bars indicate standard deviation (SD). All statistical tests were two-sided, and P < 0.05 was considered statistically significant.

## Supplementary Material

Supplementary Figure
