# Supplementary material for: Anillin is required for tumor growth and regulated by miR-15a/miR-16-1 in HBV-related hepatocellular carcinoma
Source: Aging (Albany NY). 2018 Aug 9;10(8):1884–901. doi: 10.18632/aging.101510 (PMC6128427; doi:10.18632/aging.101510)
Supplement: Supplementary Figure [file aging-10-101510-s001.pdf]

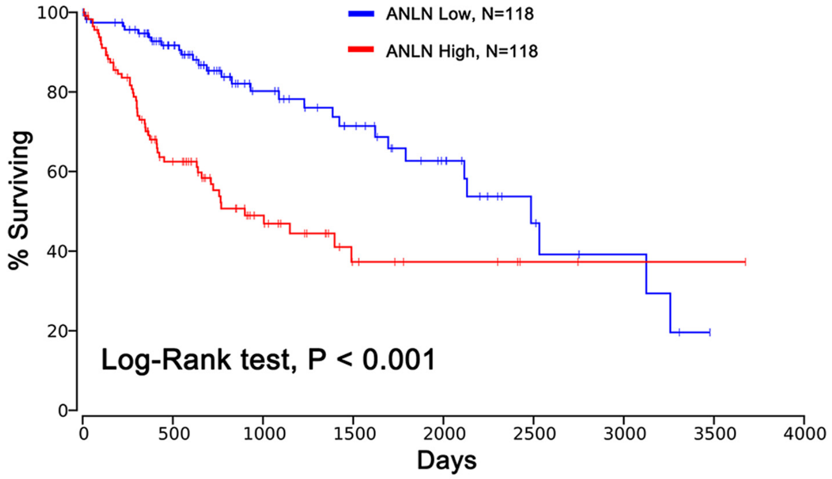

Supplementary Figure 1. ANLN expression is associated with overall survival using OncoLnc survival data according to Kaplan-Meier analysis.
